# Why girls get married early in Sarawak, Malaysia - an exploratory qualitative study

**DOI:** 10.1186/s12905-020-00911-z

**Published:** 2020-03-04

**Authors:** Ayako Kohno, Maznah Dahlui, Nik Daliana Nik Farid, Razitasham Safii, Takeo Nakayama

**Affiliations:** 1 Department of Health Informatics, School of Public Health in the Graduate School of Medicine, Kyoto University, Yoshida Konoe-cho, Sakyo-ku, Kyoto, 606-8501 Japan; 2 Centre for Population Health, Department of Social and Preventive Medicine, Faculty of Medicine, University of Malaya, 50603 Kuala Lumpur, Malaysia; 3 Faculty of Public Health, Airlangga University, Fakultas Kesehatan Masyarakat, Kampus C, Mulyorejo, Kota SBY, Surabaya, Jawa Timur 60115 Indonesia; 4 Department of Social and Preventive Medicine, Faculty of Medicine, University of Malaya, 50603 Kuala Lumpur, Malaysia; 5 Faculty of Medicine and Health Sciences, University of Malaysia Sarawak, 94300 Kota Samarahan, Sarawak Malaysia

**Keywords:** Qualitative research, Child marriage, Sarawak, Malaysia

## Abstract

**Background:**

Child marriage, a marriage that involves someone under the age of 18 years, is a long-standing social issue in Sarawak state, Malaysia. The state has taken several measures to improve situations of inequity for women who get married early; however, the practice is still a common part of the tradition and culture. The aim of this study was to explore the factors leading to child marriage in Sarawak state, Malaysia.

**Methods:**

This was an exploratory qualitative study conducted via semi-structured interviews with twenty-two women who were married when they were younger than 18 years old in Kuching, Sarawak, Malaysia. Participants were recruited through purposive and convenient sampling with the use of data from a reproductive health clinic and recruitment in villages. Thematic analysis was used for data analysis.

**Results:**

Four overarching themes were identified: health risk behaviour, family poverty, early marriage as fate, and family disharmony.

**Conclusions:**

In-depth understanding of the unique factors leading to child marriage locally will facilitate the introduction of new approaches to interventions to eradicate child marriage in Sarawak state, Malaysia.

## Background

Child marriage is a globally occurring social phenomenon in which adolescents are married when they are younger than 18 years old. The United Nations Children’s Fund defines child marriage as a formal marriage or informal union in which one or both partners are aged below 18 years old [1]. This study focused on child marriage in Sarawak state, Malaysia. Malaysia is a federal constitutional monarchy in which Islam is the majority religion, and the country’s territories encompass Peninsular Malaysia and Malaysian Borneo that include Sarawak state. According to the latest census of 2010, the rates of marriage among female youth aged 15 to 19 years in Sarawak states was 9.4%, comparing with the national rate of 6.1% [2]. For Muslims in Malaysia, Islamic Family Law (Federal Territory) Act 1984 applies, which states that the minimum age for marriage is 18 years for males and 16 years for females [3]. However, in practice, if any Muslim below the abovementioned ages in Malaysia wants to get married, he or she can do so by obtaining the consent of his or her parents or guardian and the approval of a judge in the religious court (Shariah). What makes it difficult to eliminate child marriage is because the voices of those who are affected by child marriage are difficult to hear due to their vulnerability in society. Thus, the prevention of child marriage has not been a subject of public and political debate. In Europe, there has only recently been a debate on forced marriage, including child marriage, as cases of child marriage are closely linked to migration [4].

Previous studies have reported the detrimental effects for girls in child marriages, such as lifetime poverty, low access to health care, and gender inequity. These are perceived to be cumulative factors that affect girls who get married below 18 years of age [5]. Previous studies have also reported that child marriage may result in devastating health consequences, such as human immunodeficiency virus/ acquired immunodeficiency syndrome (HIV/AIDS) and sexually transmitted diseases, cervical cancer, malaria during pregnancy and related complications [5–7]. Additionally, child marriage has been reported to be associated with risks to mothers’ and children’s health due to childbearing at a young age [6, 8, 9]. Because of the immaturity of girls who get married and become pregnant while they are teenagers, some are not physically, physiologically and psychologically ready to take on the responsibility of childbearing [6]. If a girl’s pelvic bone is too narrow, she will not be able to deliver the baby and may thus have to deliver by emergency caesarean section; obstructed labour and accompanying obstetric fistula may leave some girls with permanent damage to their health and well-being, and in the worst case scenario, maternal or infant death in childbirth may occur [6, 9]. Thus, child marriage is considered a human rights violation within the international community, by such organisations as the World Health Organization (WHO) and the United Nations. However, the factors contributing to child marriage may differ, as they are shaped by the socio-cultural contexts of each country and region.

In this study, a conceptual framework of locus of control was used. This theory was developed by Rotter who described a degree to which people believe that they have control over the outcomes of events in their lives as opposed to attributing these outcomes to external forces beyond their control [10]. According to Fiori et al., people with an external locus of control tend to believe that rewards are largely determined by external forces such as fate, luck, chance, the government, or powerful others [11]. In this study, the findings are analysed in alignment with locus of control theory, so as to unravel the complex psychological perception of the girls as to why they enter into child marriage in Sarawak state.

In order to prevent child marriage in Sarawak state, Malaysia, it is important to have an in-depth understanding of the background factors of child marriage in the local context. Therefore, the aim of this study is to explore the factors leading to child marriage in Sarawak, where child marriage is still practised according to cultural and societal norms. By examining the factors contributing to child marriage in Sarawak, we can reveal the background socio-cultural determinants that influence girls and their families in their decision to pursue child marriage.

## Methods

### Research design and rationale

This qualitative study involved semi-structured interviews with women who were married when they were younger than 18 years old in Kuching, Sarawak State, Malaysia. Critical realism was the ontological stance adopted in this study; critical realism suggests that reality can be known through the investigation of human minds and socially constructed meanings [12, 13]. This stance was adopted in this study so that the exploration could reveal the factors that affected the participants’ views towards child marriage. The data analysis was conducted using thematic analysis [14, 15]. The thematic analysis approach by Braun and Clarke was chosen because it allows researchers to conduct analyses with flexibility and by following six distinctive phases: 1) becoming familiar with the data, 2) generating initial codes, 3) searching for themes, 4) reviewing themes, 5) defining and naming themes, and 6) producing the report. The application of thematic analysis with an ontology of critical realism, can assist in analysing the experiences, meanings and reality of the study participants. Through a focus on the aim of this study, insights regarding the factors leading to child marriage could be revealed. In addition, to describe the nuanced variations within the themes, an applied thematic analysis approach proposed by Guest was used to elaborate on the comparisons of quotes within the themes [15]. This study is in alignment with the items of the consolidated criteria for reporting qualitative research (COREQ) and includes the information required in this guideline [16].

### Setting

This study was conducted at a reproductive health clinic and in villages of suburban Kuching, Sarawak State, Malaysia. This state was chosen because it has one of the highest rates of child marriage in Malaysia [2]. In the urban and suburban settings of Kuching District, the issue of child marriage seems problematic, but there is very little scientific knowledge about the phenomenon of child marriage; only anecdotal cases of child marriage have been reported through personal communications with academic researchers and healthcare workers in the area.

### Sampling and recruitment

Purposive and convenient sampling strategies were used in this study. Recruitment of the women was initiated by contacting the clients of a private reproductive health clinic in Kuching. The first wave of participants included women who were recommended by the nurses of the reproductive health clinic, who used records to identify clients who met the following inclusion criteria: Malaysian females who were first married when were younger than 18 years old, who resided in the study area during the study period, and who were of reproductive age (current age between 18 and 49 years). The candidates were contacted by the clinic nurse, and only those who agreed to participate were then contacted by the researcher. None of the women who were contacted refused to participate in this study, but we did not complete an interview with one of the candidates because we found she did not meet the inclusion criteria after we had started asking her questions at the beginning of the interview. For the second wave of recruitment, we visited households in the villages and identified women who met the inclusion criteria with the help of five local liaison officers. This process was undertaken to seek participants from diverse communities within Kuching District. The liaison officers asked the village members questions to determine their eligibility based on the inclusion criteria, and only those who met the criteria and were interested in participating in this study were approached by the researcher. We visited six different villages within the study area. This strategy of reaching out to the community was chosen so that we could effectively identify women who had married at younger than 18 years as we had discovered through an information exchange with the liaison officers that these women tended to live in several specific villages within Kuching District.

### Procedure and materials

We conducted one-to-one semi-structured interviews to explore the women’s experiences of child marriage, including their perceived reasons for child marriage and the reactions of their parents, friends and community members about their marriage at a young age. The interviews took place during July and August 2017 either in the clinic’s office for the participants who were recruited from the clinic or at the participant’s house for those who were recruited in the villages. One interview was conducted per participant, and the interviews lasted between 13 and 56 min, with an average interview time of approximately 30 min. In all cases, privacy was ensured by conducting the interviews in a private room where no other person was present except the local interviewer, the primary researcher (AK) and the participant. At the beginning of each interview, with the help of the interpreter, the primary researcher briefly explained her role of leading the study team and her background as a female Japanese researcher interested in the research topic of those who married at a young age in Sarawak and expressed her appreciation for the time the participants were taking to participate in this research. As the culture of Sarawak was foreign to the primary researcher, she tried to be respectful of the responses and behaviour of the participants and at the same time, to maintain an explorative approach to understand the unique culture of Sarawak. For that purpose, the primary researcher strategically used reflexivity, a continual internal dialogue and critical self-evaluation, as the quality control method in this study [17]. The primary researcher took field notes during and after the interviews to describe the living conditions of the women as well as other specifics observations during the interview, such as the characteristics of the participants and the surrounding environment in the community. As we visited the houses of the participants in the villages, the primary researcher was able to directly observe the living conditions of the participants and their interactions with family members and neighbours, and the primary researcher wrote her perceptions and reflexive thoughts in field notes. Interviews were conducted with women of diverse ethnic and religious backgrounds to ensure the validity of the study findings [18]. The interviews were conducted until the point of data saturation, i.e., “when no new categories or concepts can be derived from the data and any further data collected will fit within already developed categories” [19]. In this study, data saturation was reached after interviewing 20 women, and an additional two interviews were conducted to confirm that data saturation had been reached. A semi-structured interview guide had been previously prepared, and it was used during the interviews as a reference guide. The guides consisted of questions about what the women thought were the main reasons leading to their marriage at a young age as well as the reactions of their family, community, and friends. The participants were also asked about their current and past health statuses, childbearing experience and contraceptive use. Other probing questions that were not written on the guide were also asked when necessary to gain more insights on the issues discussed by the participants. As the interview guide had previously been developed for and used in a qualitative study conducted in another region in Malaysia [20], no pilot test was conducted. English version of the interview guide was published in abovementioned publication as the online supplementary documents. Interviews were conducted in the Malay language by a local research assistant who had sufficient knowledge of the local cultural and religious aspects. Prior to this study, the research assistant had experience as an interviewer in another qualitative study and received 10 h of training to gain skills in interviewing, before we commenced the interviews. The interviews were audio-recorded with the prior permission of the participants after the purpose and summary of the study were explained. The recorded data were transcribed into the Malay language and translated into English by a professional translator.

### Data analysis

A thematic analysis was conducted wherein patterns or themes within the data were analysed and reported using the following six phases: becoming familiar with the data, generating initial codes, searching for themes, reviewing themes, defining and naming themes and producing the report [14]. In addition, we compared the data among the following three groups according to the current age of the women to show the nuanced commonalities and differences within each theme by using the applied thematic analysis approach by Guest [15]: younger age group (current age: 18–25 years), middle-aged group (26–35 years), and older age group (more than 35 years old). First, the primary researcher familiarised herself with the data by reading the interview transcripts three times. In addition, field notes were studied to incorporate the primary researcher’s observations during the analysis process. During the initial coding process, segments of the transcripts were assigned with a brief description of the meanings and phenomena using NVivo version 11 software (QSR International, Australia) for data management. After that, the codes were sorted into potential themes. Additionally, the relationships between the main themes and sub-themes were considered. Then, the quoted data were tabulated according to the three age groups. These themes were then refined through a review of the coded data extracts to determine whether the assigned themes articulately portrayed the meanings that were evident in the whole data set. Subsequently, the names of the themes were examined and finalised to fully reflect the stories behind each theme. Finally, the results of the analysis were reported in a written extract of the stories revealed by the analysis process. The primary researcher created the initial version of the coding table that contained the list of codes, sub-themes and themes that the three researchers (AK, MD and ND) identified. Divergences of opinions were discussed and reconciled, and the coding table was modified accordingly through a series of discussions. The participants’ verbatim comments are presented as part of the findings. Each comment is marked with a specific identification number that was assigned to each participant to protect her anonymity (e.g., No 1, ethnicity, age at marriage, current age group at the time of interview). Current age group is categorized into the following three: Group 1 - women whose current age was between 18 and 25 years at the time of interviews; Group 2 - women aged 26 to 35 years; and Group 3 - women aged above 36 years.

## Results

### Participants

This study included 22 women who had experienced child marriage. The participating women had a mean age of 30.6 years and a mean marriage age of 16.3 years. The socio-demographic characteristics of the women are shown in Table 1.

**Table 1:** Socio-demographic characteristics of women who experienced child marriage (*n* = 22)

Characteristics	Range in Years (Mean)	Data by Age Group		
		Group 1^a^ (*n* = 8)	Group 2^a^ (*n* = 8)	Group 3^a^ (*n* = 6)
		Frequency (Percentage)		
Current Age	18–46 (30.6)	8 (36.4%)	8 (36.4%)	6 (27.3%)
Age at Marriage	14–17 (16.3)	16–17 (16.6)	15–17 (16.4)	14–17 (16.0)
Current Marital Status	Frequency			
Continued First Marriage	19	7	7	5
Divorced and Remarried	2	1	1	0
Widowed	1	0	0	1
Final Education Attainment	Frequency			
Primary School	2	0	0	2
Lower Secondary (Forms 1–3)	8	4	4	0
Upper Secondary (Forms 4 and 5)	11	3	4	4
Diploma	1	1	0	0

^a^ Group 1: Women whose current age was between 18 and 25 years

Group 2: Women aged 26 to 35 years

Group 3: Women aged above 36 years

Four overarching themes with respect to the factors leading to child marriage were identified: health risk behaviour, family poverty, early marriage as fate, and family disharmony. For each theme, we compared the coded quotes for each subtheme among the three age groups to show the nuanced commonalities and differences within a given theme by age group. Tables 2, 3, 4 and 5 show exemplar quotes for each theme by age group.

**Table 2:** Comparison of the age groups within each subtheme (theme: health risk behaviour)

Group 1 (18–25 years)	Group 2 (26–35 years)	Group 3 (> 35 years old)
Subtheme 1: Unprotected Intercourse and Pre-marital Conception		
“Actually I...got pregnant before marriage. So, we got married. Actually, we were planning to get married even before the pregnancy anyway.” (No. 5, Malay, married at 17 years old.) “Because I was 3 months pregnant (outside of marriage). I told her (grandmother) I didn’t want to (get married). My grandmother on my father’s side gave me two choices. First, she would send me to the city to study, and she told me to abort the baby. But then I didn’t want to. I had sinned once (by becoming pregnant outside of marriage); I didn’t want to commit another one.” (No. 6, Malay, married at 17 years old.) “I was pregnant. I had sex outside of marriage.” (No. 12, Malay, married at 17 years old.) “(I got married) because I was pregnant at that time. I didn’t want it (marriage) at first. But what could I do; I’ve gone overboard.” (No. 14, Malay, married at 16 years old.) “How do I say this... when I was pregnant with the first child, I wasn’t.... when I was pregnant, I wasn’t married yet.” (No. 23, Malay, married at 16 years old.)	“I was in the middle of Form 4 (at 16 years old) and then I quit school. Because, um, pregnant. Because I was pregnant, I could not finish my studies.” (No. 4, Malay, married at 16 years old.)	We had sexual relations (before marriage) then, even though it was wrong. Then I found out about the pregnancy at the time when I was working.” (No. 1, Malay, married at 15 years old.) “When we were young we didn’t plan to get married, but at that time we were caught doing indecencies. So according to Islam, we had to get married.” (No. 15, Iban, married at 17 years old.) “Well, when I was young, I had pre-marital sex. So, we had to get married. Our parents told us to get married as well, so it was better to get married.” (No. 17, Malay, married at 17 years old.)
Subtheme 2: Alcohol and Drug Misuse		
After I quit school, I did nothing but just enjoyed myself.... First, I was in jail because my mom was suspicious about my behaviour, and I always talked back. So, she wanted to check, and she told the police to take me. They checked my urine and took me to the drug rehabilitation centre.” (No. 5, Malay, married at 17 years old.) I... always went out; I was naughty. Followed my friends, drank alcohol... I got into a fight with my grandmother, and I ran away.” (No. 6, Malay, married at 17 years old.) “I had many friends who were tomboys, many of them. So, my father, when he found out that I was friends with them, because the majority of them drank alcohol and everything, so he was worried that I would drop out of school because of them. When we’re friends with someone, we tend to do what they do. So, my father found... a Nospen pill (a type of drug) in my pocket, but it was not mine.” (No. 22, Malay, married at 17 years old.)	(No relevant quotes.)	(No relevant quotes.)

**Table 3:** Comparison of the age groups within each subtheme (theme: family poverty)

Group 1 (18–25 years)	Group 2 (26–35 years)	Group 3 (> 35 years old)
Subtheme 1: School Dropout		
“I wanted to quit (school). No one made me do it. I was wild at that time.” (No. 5, Malay, married at 17 years old.) My husband was one (of the reason to quit school), and I was pregnant also. (No. 6, Malay, married at 17 years old.) “(I quit school) because I wanted to get married. No one asked me to quit.” (No. 9, Malay, married at 16 years old.) “I quit school and worked in a canteen. I myself wanted to quit because my parents could not afford it anymore.” (No. 12, Malay, married at 17 years old.)	“I was in the middle of Form 4 (at 16 years old) and then I quit. Because um... pregnant.” (No. 4, Malay, married at 16 years old.) “I quit school because I got married.” (No. 11, Malay, married at 16 years old.) “Because we only lived a simple life; my parents were unemployed, my brothers worked but were not so rich, and then I decided to quit school when I was in Form 1 (at 13 years old).” (No. 13, Malay, married at 16 years old.) “I quit school because I had to work after my mom died.” (No. 19, Malay, married at 15 years old.) “I was too lazy to go to school. At that time I was waiting... (I had) the PMR (Lower Secondary Assessment examinations) at that time, and I worked. I liked working, and I’ve lost interest in studying. Besides that, our lives were difficult back then. I had many siblings.” (No. 20, Malay, married at 17 years old.)	“(I quit school and) worked at a restaurant. I wanted to work. When I was little at the time, I was too lazy to study and it wasted my parents’ money.” (No. 1, Malay, married at 15 years old.) “[It was] my aunt’s choice (for her to stay with her while going to school) because my parents wanted me to quit school. My siblings were still too little; we were villagers.” (No. 2, Bidayu, married at 16 years old.) “I quit when I was in Standard 6 (at 12 years old). My parents could not afford it.” (No. 7, Iban, married at 14 years old.) “I was still a student, but because of pre-marital sex, and because he agreed as well, we just got married. I was in the middle of school.” (No. 17, Malay, married at 17 years old.)
Subtheme 2: Reducing the Burden on Parents		
“I thought that my marriage would not burden my parents because my husband is able to take care of me, and we do not have to ask money from my parents anymore.” (No. 12, Malay, married at 17 years old.)	“After I quit (school) I wanted to continue, but then I pitied my parents, so I couldn’t.” (No. 13, Malay, married at 16 years old.) “I quit school when I was 15 years old because I worked after my mom died. She was sick. Because at that time, my mom was sick and my dad didn’t have a stable job. I have brothers, but all of them were already married.” (No. 19, Malay, married at 15 years old.) “Sometime after 10 years old, I went back to my mother’s house. At that time someone was looking for a nanny to take care of their children. So, my mother said, ‘You have no job, so it’s better if you work now,’ she said. So, my sister and I agreed to take the job. Both of us became a nanny to take care of the children.” (No. 20, Malay, married at 17 years old.)	“(I quit school and) worked at a restaurant. I wanted to work. When I was little at the time, I was too lazy to study, and it wasted my parents’ money.” (No. 1, Malay, married at 15 years old.) “Because we were in a difficult life, and when we get married, the husband will pay for everything. So, we have an open mind to, as they say, when, how to say this, we won’t burden our parents too much (by getting married) because I have four siblings, I got married and then there are three, and my brother lived on his own. So, my parents had fewer burdens and could send my younger siblings to school. I, too, after I got married, I could help my siblings. I bought them clothes, trousers, and a little food. At the end of the month, I sent them money too. I got married, and my parents’ lives got easier.” (No. 7, Iban, married at 14 years old.)

**Table 4:** Comparison of age groups within the theme (theme: early marriage as fate)

Group 1 (18–25 years)	Group 2 (26–35 years)	Group 3 (> 35 years old)
“I don’t know why (I got married). Maybe it’s fate.” (No. 9, Malay, married at 16 years old.)	“There were (people talking behind my back), but I ignored them. Because it’s already fate.” (No. 4, Malay, married at 15 years old.) “I don’t know how it led to marriage. It’s fate, as they say. We stayed married until now.” (No. 8, Malay, married at 17 years old.) “I guess it’s fate. Nobody forced us (to get married)” (No. 11, Malay, married at 16 years old.) “Maybe it’s fate (that I got married), and our families have given their blessing.” (No. 16, Malay, married at 17 years old.)	“At that point, I viewed young marriage as my fate, but it wasn’t well-received by my family because I was still a child.” (No. 2, Bidayu, married at 16 years old.) “It’s normal. That’s what they call fate.” (No. 3, Malay, married at 17 years old.) “(The ideal age of marriage is in your) 20s. But what to do? It’s my fate.” (No. 17, Malay, married at 17 years old.)

**Table 5:** Comparison of age groups within the theme (theme: family disharmony)

Group 1 (18–25 years old)	Group 2 (26–35 years old)	Group 3 (> 35 years old)
“My parents were divorced when I was in Primary 4 (at 10 years old).” (No. 5, Malay, married at 17 years old.) “I lived with my grandmother since I was 4 years old until Form 5 (17 years old).” (No. 6, Malay, married at 17 years old.)	“My father married my mother before he got divorced and married to another, and then divorced again, and now he has another wife.” (No. 8, Malay, married at 17 years old.) “My parents had their problems. They got divorced when I was in Standard 4 (at 10 years old); they separated.” (No. 16, Malay, married at 17 years old.) “I was under my grandmother’s care since I was young. I only went back to my mother’s house when I was older.” (No. 20, Malay, married at 17 years old.)	“My father had proposed a potential husband for me, but I didn’t want him. I didn’t want a man who was already married and looking for another wife to convert to Islam. So, I ran away. I looked for a job so I wouldn’t have to marry. I found my own money first. When I was old enough, I was pressured to marry. He (the father) told me to marry that old Chinese man. I didn’t want to. So, I had to find a young man.” (No. 1, Malay, married at 15 years old.)

### Health risk behaviour

This theme consisted of two subthemes: unprotected intercourse and pre-marital conception and alcohol and drug misuse.

#### Unprotected intercourse and pre-marital conception

For many of the participants, the turning point in making the decision to marry as children was when their parents suspected them of either having pre-marital sex or “khalwat” (close proximity) or discovered that they were pregnant before marriage, all of which are prohibited in their religion, Islam. Due to religious norms and to conceal the shame and disgrace of the family, the parents in these situations forced the girls to get married immediately, as the girls were thought to have committed sins.

“Well, when I was young, I had pre-marital sex, so we had to get married. Our parents told us to get married as well, so it was better to get married. So, we fell in love only after we got married to each other. Before marriage, we just liked each other. My husband’s mother told us to get married because of pre-marital sex. I was still a student, but because of pre-marital sex and because he agreed as well, we just got married. I was in the middle of school. My father’s side of the family told me that instead of letting a bad thing (pre-marital sex) continue, it’s better to get married.” (No. 17, Malay, married at 17 years old, current age range in Group 3.)

“There were many obstacles prior to our marriage. We had sexual relations (before marriage) then, even though it was wrong, and then I found out about my pregnancy at the time when I was working. My boss told me to quit because once I was pregnant, I could not work for long hours. I confronted my father. At first, he was furious. He thought that if he didn’t marry us immediately, the child would be born outside of wedlock, and that is a sin.” (No. 1, Malay, married at 15 years old, current age range in Group 3.)

“I was in the middle of Form 4 (at 16 years old) and then I quit school. Because, um, pregnant. Because I was pregnant, I could not finish my studies. Ah, after I found out I was pregnant, I quit immediately. We married. If I hadn’t been pregnant, I wouldn’t have wanted to marry. I would have continued studying. Because while I was checking (for the pregnancy), both of our families discussed it; they said if I hadn’t been pregnant, I would have been engaged first. After I finished Form 5 (at 17 years old), I would have gotten married because I had to finish my studies first. However, because I was pregnant, they... I was told to get married.” (No. 4, Malay, married at 16 years old, current age range in Group 2.)

In Sarawak, where people of diverse ethnicities reside together, the Islamic teaching on the prohibition of pre-marital courtship may also be applicable to non-Muslims if their partners are Muslim. An Iban woman (belonging to one of the native ethnic groups of Borneo Island) who had married a Muslim man explained that she married at an early age because they were in situation of “khalwat” (close proximity).

“When we were young we did not plan to get married, but at that time, we were caught doing indecencies. So, according to Islam, we had to get married. It’s like we were dating alone, just the two of us. We weren’t supposed to go out after 12 o’clock.” (No. 15, Iban, married at 17 years old, current age range in Group 3.)

Notably, most of the women in this study stated that they did not have proper knowledge of how women could become pregnant prior to their early marriage. They simply did not know about the biological mechanism of becoming pregnant before they had intercourse at a young age.

“I think I had sex when I was in Form... still in school, Form 4 (at 16 years old). Two or three months before my marriage, that was my first time. After having sex, I immediately got pregnant. Never. I never wanted to. I asked what, like one of my friends, she said that her boyfriend touched her breast; I asked my friend ‘If he touched your breast, can you get pregnant?’ She said no, only if our genitals touched, she said. I was like, ha, okay and a month or two months after that I didn’t get my period. So, my mom became suspicious because I didn’t ask her to buy pads, so she was suspicious because I had not asked her to buy them for almost two months. So, she suspected it and brought me to the clinic. Ha like that.” (No. 4, Malay, married at 16 years old, current age range in Group 2.)

#### Alcohol and drug misuse

Some of the women in this study related that they were involved in risky activities, such as consuming alcohol and drugs, with their peers when they were adolescents. Through the network of friends that they spent time with, they found partners with whom they had sexual relations and became pregnant. Although these women stated that the pregnancy was the triggering event for the decision to get married early, they also explained that they had actually wished to be married so that they could fill the void of loneliness, as they believed that by getting married, they could change their lives and not be involved in alcohol and drug use anymore.

“After I quit school, I did nothing but just enjoyed myself. I was more into enjoying myself. First, I was in jail because my mom was suspicious about my behaviour, and I always talked back. So, she wanted to check, and she told the police to take me. They checked my urine and took me to the drug rehabilitation centre. So, I was thrown into the (drug rehabilitation) centre. But even after I was released, my behaviour didn’t change. I was stubborn. After a while, I was tired of being bad, so I thought I should just get married. For me, I thought it would make my parents not worry anymore if I got married. If I didn’t have a husband, then they would be constantly worrying about me. I was very wild. I was like a crab, as they say; I always went out somewhere. Alhamdulillah (thank god), now that I have a husband, I am okay; I can change on my own.” (No. 5, Malay, married at 16 years old, current age range in Group 1.)

“I... always went out, I was naughty. Followed my friends, drank alcohol... I got into a fight with my grandmother, and I ran away. I was contacted by the welfare people again and then they said, ‘Because you keep running away, we will send you to the girls’ home.’ Because I kept on running away from home. Unless someone wanted to take me in. So, my grandmother said, ‘Do you want to follow me?’ I didn’t want to go to the girls’ home; I didn’t want to. If it wasn’t for my grandparents’ help, I don’t think I could have completed my studies. They took care of me since I was small. I decided to get married because I was three months pregnant. First, I told my grandmother that I didn’t want to. She gave me two choices. First, she would send me to the city to study, and she told me to abort the pregnancy. But then I didn’t want to. I had sinned once (by becoming pregnant outside of marriage); I didn’t want to commit another one. So...” (No. 6, Malay, married at 17 years old, current age range in Group 1.)

Table 2 shows the comparison of the age groups within the subthemes of health risk behaviour. The unprotected intercourse and pre-marital conception subtheme was especially evident among group 1 (18–25 years) and group 3 (above 35 years old). The alcohol and drug misuse subtheme was identified only among the youngest participant group.

### Family poverty

Under the theme of family poverty, we identified two subthemes: school dropout and reducing the burden on parents.

#### School dropout

Many of the women in this study dropped out of school long before they engaged in child marriage, primarily due to family poverty. There was also another group of women who had dropped out of school at a young age due to pregnancy out of wedlock. Several of the participants in this study stated that they left school at an early age, such as when they were 12 or 13 years old, either voluntarily due to their families’ financial situations or because they had been explicitly told by their parents to terminate schooling because of the family’s poverty. After leaving school, some girls helped their families financially by working, while others stayed at home and helped with household chores and taking care of younger siblings. The other predominant reason for leaving school was pregnancy. The decision to leave was quickly enforced by the parents after they discovered the pregnancy. After dropping out of school, one of the participants was quickly married off to the man with whom she had had pre-marital sex.

“I didn’t study at the time of my marriage. I attended school only until Primary 6 (at 12 years old). I stopped because my parents couldn’t afford it. My parents didn’t work; they only planted wheat in a long house.” (No. 7, Iban, married at 14 years old, current age range in Group 2.)

“I did not finish studying because we only lived a simple life; my parents were unemployed, my brothers worked, but were not so rich, and then I decided to quit school when I was in Form 1 (at 13 years old). After I left, I wanted to continue, but then I pitied my parents, so I didn’t. My parents were okay with me quitting school; they didn’t really care. Then, my marriage was arranged by our parents when I was 16 years old.” (No. 13, Malay, married at 16 years old, current age range in Group 2.)

“I studied until Form 2 (at 14 years old). I wanted to quit because my parents couldn’t afford it. The reaction of my parents, they were just okay with it as long as I wanted to work after I quit school.” (No. 12, Malay, married at 17 years old, current age range in Group 1.)

#### Reducing the burden on parents

Some of the participants stated that their main reason for getting married early was to help reduce the financial burden on their parents. Their logic was that by getting married early, there would be fewer members in the family for their parents to take care of. The girls thought getting married early would be beneficial for the family because it would reduce the burden on their parents.

“Because we were in a difficult life, and when we get married, the husband will pay for everything. So, we have an open mind, as they say, when, how to say this, we won’t burden our parents too much (by getting married) because I have five siblings. So, my parents had fewer burdens and could send my younger siblings to school. I, too, after I got married, could help my siblings. I bought them clothes, trousers and a little food. At the end of the month I sent them money too. I got married, and my parents’ lives got easier.” (No. 7, Iban, married at 14 years old, current age range in Group 3.)

“I wanted to quit school because my parents couldn’t afford it anymore. Then, I met my husband at my workplace, at the canteen. Then, I was pregnant, so I decided to get married. First, I felt scared that I couldn’t take care of my husband completely. Scared that I wouldn’t be able to give him food and cook for him. But I thought that my marriage would not burden my parents because my husband is able to take care of me, and we do not have to ask money from my parents anymore.” (No. 12, Malay, married at 17 years old, current age range in Group 1.)

Table 3 shows the comparison of the age groups within the subtheme of family poverty. The school dropout subtheme was identified across all age groups, while the reducing the burden on parents subtheme was identified more in the older age groups.

#### Early marriage as fate

Most of the women in this study described getting married early as their fate. They could not find any other justifiable reasons for their decision to marry early but believed that fate dictated that they would be married at an early age.

“I guess why I got married early is because it is my fate. We loved each other. Some of my friends asked me why I decided to get married too early. They said that I was young and my future’s still long; that I needed to enjoy myself. But it’s fate; that’s all I can say.” (No. 11, Malay, married at 16 years old, current age range in Group 2.)

“I don’t know why I got married early. Maybe it’s fate.” (No. 9, Malay, married at 16 years old, current age range in Group 1.)

Table 4 shows the comparison of age groups within the fate of an early marriage theme. Fate was referred to by the participants in the older age groups, who had a longer time since their marriage to contemplate on what had happened.

### Family disharmony

#### Family disharmony

Relationship problems with parents seemed to indirectly influence the women in their decisions to get married at a young age. Some of the women who participated in this study confessed that their relationships with their parents were strained during their childhood or adolescence, and that they sought affection and comfort in early marriage instead.

“My parents had their problems. They got divorced when I was in Standard 4 (at 10 years old); they separated. So, I stayed with my mom; my dad remarried. My step-mother never took care of us. She didn’t really like us, so she never objected to my marriage. It’s all up to my mom. I only took my dad as ‘wali’ (the bride’s representative in a Muslim wedding).” (No. 16, Malay, married at 17 years old, current age range in Group 2.)

“I lived with my grandmother since I was 4 years old until Form 5 (at 17 years old). When I was in Form 3 (at 15 years old), I got into a trouble; I couldn’t take it. I was under a lot of pressure. I really didn’t receive enough love from my parents since I was little. I was always with my grandmother. So, I ran away from there... it was examination time. I took my identification card and left (my grandmother’s house). I sought out my mother again because I really loved her.” (No. 6, Malay, married at 17 years old, current age range in Group 1.)

“I was under my grandmother’s care since I was young. I only went back to my mother’s house when I was older. I don’t remember when I returned to my mother’s house, but I can only recall that when I was very young; my grandmother took care of me. Sometime after 10 years old, I went back to my mother’s house.” (No. 20, Malay, married at 17 years old, current age range in Group 2.)

Table 5 shows the comparison of the age groups within family disharmony theme. Participants in the older age group mentioned this theme more than those in the youngest group.

## Discussion

In this study, we interviewed women who were married at younger than 18 years of age in Kuching, Sarawak, Malaysia. They provided in-depth information about their lived experiences regarding what led them to get married at a young age. We identified four overarching themes regarding the factors leading to child marriage in Sarawak. The themes were health risk behaviour, family poverty, early marriage as fate, and family disharmony. In this discussion section, we focus on the emotional aspects from the participants’ perspectives and elaborate on what influenced the women in this study to decide to get married at an early age.

Regarding the first theme of health risk behaviour, which included the unprotected intercourse and pre-marital conception subtheme, an unmarried pregnant woman is seen as having committed a moral violation in Malaysia, and people perceive unmarried teenage mothers as having committed delinquency by having unmarried sexual relations or practising substance abuse or prostitution [21]. Therefore, parents or guardians will try to get them married immediately if they know about the pregnancy. Compared with parents’ motives reported in previous studies of child marriage in developing countries in South Asia and Africa, the parents’ motives in this study were slightly different. The parents believed they were left with no choice but to allow their daughters to get married ex post facto due to pre-marital conception. In contrast, the motives of the parents in the developing countries mentioned above were to protect their daughters from sexual assault or prostitution that may have occurred if their daughters remained unmarried; thus, the parents felt more secure once their daughters were married and under a husband’s protection [7]. Another parental motive is that having a girl in a family is a financial burden, and thus, they need to find a suitor as early as possible [22]. In this study, we found that in most cases, the parents pressured their daughters to get married before they were 18 years old only after they had found out that they were pregnant. According to Islamic teaching, under some circumstances, when a man and a woman are together in private, they can be suspected of engaging in immoral activities that are considered “khalwat”(close proximity) and can be subject to criminal investigation under the Islamic religion [23]. For one of our participants, this was the stated reason for her child marriage; her parents had found out about her participating in an indecent act in private with her future husband and suspected that the couple was engaging in sexual behaviour. Therefore, we can conclude that child marriage in Sarawak is perceived as a mechanism for preventing young couples from committing further sins and ensuring that a girl who becomes pregnant does get married. Furthermore, one of the pertinent findings in this study is that many of the girls who became pregnant did not have adequate knowledge of how to prevent pregnancy. This finding is in line with a previous study of women who experienced early marriage in Iran [24]. Therefore, to prevent and reduce unwanted pregnancy among adolescents, sex education, including information about contraception methods, for female adolescents at school and in the community must be promoted at an early age as they experience puberty. The previous review study identified a lack of knowledge about sexuality and reproductive health among Malaysian adolescents [25]. Therefore, education must emphasise what may happen when a couple has a sexual relationship as well as the various responsibilities attached to marriage and childbearing. The challenges faced by other Islamic states, such as Iran and Indonesia, concerning the introduction of sex education at school are based on the idea that teaching about sexuality is a taboo [26, 27]. A previous study reported that there is a reluctance to discuss sexual issues in public in Iran, as it is perceived as embarrassing to talk about sexuality, and people worry about the negative impacts of introducing sexual education at school [26]. Second, the findings showed that the concept of fate affected the emotions and minds of the women and influenced their decisions to marry early. This notion of fate as one of the determinants of child marriage has been briefly reported in previous studies [28, 29] but has not been analysed in depth. In this study, the women who married at younger than 18 years of age seemed to believe that external forces determined their early marriages and that there was nothing they could have done to alter the courses of their lives. By using locus of control as a conceptual framework of this study, we can explain the fate-believing characteristic of the women in this study. Previous studies have focused on the connections between religiosity and life satisfaction, with locus of control as the mediator [11, 30]. Based on the assumption that the women in this study made decisions about child marriage within the framework of an external locus of control, it can be hypothesised that their decisions to marry early were justified and affirmed by their beliefs that it was their fate to get married at a young age. In a previous study on child marriage, a belief in fate was described as a way for married girls to quickly secure their identities, statuses, and respect in a new environment after marriage [6]. Another report described the older generation’s perception that the acceptance of early marriage as fate demonstrated girls’ submissiveness [31].

Third, it is important to closely examine the factors related to family disharmony and elaborate on how the women’s relationship problems with their parents influenced their decisions to marry as children. As yet, this issue of family disharmony has not been investigated in studies of child marriage. The salient underlying condition is the problematic relationship with parents, triggered by divorce or a broken family. One study described the typical parenting style in Malaysia as authoritarian [32]. This means that parents in Malaysia tend to be “highly controlling and demanding but affectively cold, requiring children to be responsive to parental demands” [32]. While we cannot make a generalised statement about the parenting styles of the parents of the participants in this study, it is important to further investigate the possible influence of the parent-child relationship style when examining the issue of child marriage. From the daughters’ perspectives, we can hypothesise that due to the divorce of their parents, the adolescent girls lacked affection and thus sought it elsewhere by developing relationships with men, or by marrying at a young age as a way to secure affection and emotional comfort. Furthermore, if their parents’ parenting style were authoritative, then the girls’ perceived lack of affection as a result of their parents’ divorce may have been exacerbated and consequently have further motivated them to seek refuge in child marriage. Although one study focused on the risk of child marriage and early sexual behaviours among female orphans in 10 sub-Saharan African countries [33], no studies regarding the relationship between parenting style or the absence of parental affection and child marriage have been conducted, and further investigation is needed.

Finally, we need to pay close attention to the issue of alcohol and drug misuse, which contribute to child marriage. Some of the women in this study had already been involved in risky behaviours, such as alcohol and drug consumption, during early adolescence prior to their marriages. They indicated that their sexual experience before marriage with delinquent partners occurred under the influence of alcohol and drugs. Choon et al. reported on the situation of juvenile delinquency in Malaysia, stating that when adolescents are attached to their peers rather than their parents, they are more likely to be involved in delinquent behaviours [34]. We found that some of the women in this study who were involved in delinquent behaviours during adolescence did not receive enough affection from their parents and actively sought to establish bonding relationships with delinquent male peers; they thought that an early marriage would give them the opportunity to escape from the influence of other delinquent peers. They were tired of being continuously involved in delinquent activities and wanted to find a way out of such situations. Under such circumstances, child marriage was one of the most feasible options for them. This finding is in line with a previous report from a database study examining the adult outcomes of adolescent girls who engaged in antisocial behaviour, which reported a trend towards early marriage among girls with antisocial behaviour [35]. Furthermore, a study in the field of criminology showed an association between marriage and crime in early adulthood. This study demonstrated that some women with previous records of offences seemed to be influenced by marriage and that marriage could work to suppress offending behaviour by establishing informal social control [36]. It also revealed that women who were involved in alcohol and drug misuse in early adolescence had motive to get married at a young age, to stop their involvement in risky activities and exchange their existing lifestyle for better conditions. Laub, Nagin and Sampson, using a dynamic statistical model analysis and longitudinal data, reported on the issue of desistance from crime and how early marriage prevented criminal activities among some male respondents with a history of criminal activities [37]. These researchers added that although marriage could be a turning point, desistance from crime is a gradual process that requires the accumulation of social bonds, such as attaining relationships of enduring attachment as a result of entering into a marriage or continued employment in the labour market [37]. We can apply this perspective to our study findings and argue that child marriage could provide a turning point that allows girls to leave behind habits of juvenile delinquency and that supports their desistance from criminal activities. However, this hypothesis requires careful examination through further studies.

### Future directions and implications

The findings of this study have several implications for future research on the topic of child marriage prevention. First, our findings indicate that unwanted pregnancy is a triggering factor in the decision to marry early in Malaysia. To reduce unwanted pregnancy, the implementation of sex education in early adolescence is crucial. It is important to conduct research to evaluate the effectiveness of sex education as an intervention programme at school and in the community. An innovative approach that incorporates sex education in religious teachings is the key to success. A qualitative study conducted among young Muslim women in Australia revealed the importance of aligning their sexuality with both traditional expectations and mainstream norms in Australian society [38]. This alignment was necessary to reflect the norms and expectations of both spheres. This finding gives insight into the use of sex education to prevent child marriage. We need to disseminate information and provide space for dialogue between community members and young girls about sexuality in a way that is culturally and religiously sensitive so that young girls can feel safe and secure in learning about sexuality without facing the struggle of balancing two opposing values. Second, our findings suggest the need to solve the problem of family poverty and school dropout due to poverty. Therefore, the government should enhance efforts to empower people to improve their economic statuses and promote their health literacy. A study focusing on the challenges of poverty reduction policy in Malaysia addressed the inadequacy of the capacity building of economically vulnerable groups [39]. There is a gap in educational achievement among those who are economically advantaged and disadvantaged. The implementation of programmes to scale up practical, on-the-job training for economically vulnerable groups is necessary. To create a continuous improvement loop, the effects of such interventions must be measured systematically in future studies. Third, the study shows that the parent-daughter relationship was problematic for some of the participants. The emotional impact of parents’ divorce or a child’s separation from the family due to family breakdown must be studied in connection with girls’ decisions to marry as children. One study found an association between the daughter’s satisfaction with her relationship with her mother, the mother’s strong disapproval of her daughter having sex, and the frequency of the mother’s communication with the parents of her daughter’s friends with later sexual debut [40]. In future research focusing on parent-daughter relationships and child marriage, we need to further investigate the aspects mentioned above.

### Strengths and limitations

This research is the first community-based study to reveal the factors leading to child marriage in Sarawak. The study participants were recruited from urban and suburban areas of Sarawak State; therefore, the results cannot be generalised to rural settings in Sarawak, where different factors may have a predominant role in encouraging child marriage. In this study, the factors leading to child marriage were explored from the women’s perspectives but not from the perspectives of men or the parents of those who married early. This approach might have limited the findings in terms of reflecting the viewpoints of those involved in child marriage. As one of our findings concerned family disharmony, the perspectives of parents regarding their daughter’s early marriage should be investigated in future research. We also collected data from stakeholders who interact closely on regular basis with the girls who get marry at a young age. However, due to lack of resources, we were not able to obtain data from the stakeholders until the point of saturation. Therefore, the data was not included in this study. The authors plan to publish another paper separately as a future study to focus on the perspectives of the stakeholders in the community in perceiving why child marriage take place in Sarawak. In addition, because of the sensitivity of the issue, it was not possible to collect data about the reasons for child marriage from the girls before they enter into child marriage. Despite these limitations, the findings of this study can provide an evidence base to allow policy makers and practitioners to untangle the complex array of factors that affect child marriage in Sarawak.

## Conclusions

The findings of this study show that the factors leading to child marriage in Sarawak, Malaysia are related to individual personalities as well as relationships with parents and peers. These findings can be used by policy makers when creating intervention programmes targeting young female adolescents to strengthen sex education and empower female adolescents so that they will not choose child marriage but will find hope in other meaningful life goals. Strengthening sex education for female adolescents at school and in the community as early as the onset of puberty is necessary to prevent and reduce unwanted pregnancy. Additionally, it is important to involve family members in sex education at school so that a close understanding between the family and the school can be achieved. Solely raising the legal minimum age of marriage will not solve the problems of child marriage as the factors leading to child marriage are diverse and intricately intertwined and affect the lives of girls, girls’ families and the society in which girls live. It is also important to create support programmes for girls in poor families so that dropping out of school early is not an option. Future studies are required to examine the association of child marriage with personality and with interpersonal domains such as belief in fate, school dropout and relationships with parents and peers.

## Data Availability

The datasets generated and/or analysed during the current study are not publicly available to protect the anonymity and confidentiality of the participants.

## Abbreviations

COREQ: COnsolidated criteria for REporting Qualitative research
HIV/AIDS: Human Immunodeficiency Virus/ Acquired ImmunoDeficiency Syndrome
WHO: World Health Organization
